# Insights into the theranostic value of precision medicine on advanced radiotherapy to breast cancer

**DOI:** 10.7150/ijms.49544

**Published:** 2021-01-01

**Authors:** Man Jiang, Jianshe Yang, Kang Li, Jia Liu, Xigang Jing, Meiqin Tang

**Affiliations:** 1 3 rd Affiliated Hospital of the Chinese University of Hong Kong (Shenzhen), Shenzhen 518172, China.; 2 Department of Oncology, Longgang District People's Hospital, Shenzhen 518172, China.; 3 Medical College of Wisconsin (Milwaukee), Wisconsin 53226, USA.; 4 Department of Hematology, Longgang District People's Hospital, Shenzhen 518172, China.

**Keywords:** breast cancer, radiotherapy, precision medicine, radiotherapy regime, radiosensitivity

## Abstract

Breast cancer is the most common cancer in women worldwide. “Breast cancer” encompasses a broad spectrum of diseases (i.e., subtypes) with significant epidemiological, clinical, and biological heterogeneity. Each of these subtypes has a different natural history and prognostic profile. Although tumour staging (TNM classification) still provides valuable information in the overall management of breast cancer, the current reality is that clinicians must consider other biological and molecular factors that directly influence treatment decision-making, including extent of surgery, indication for chemotherapy, hormonal therapy, and even radiotherapy (and treatment volumes). The management of breast cancer has changed radically in the last 15 years due to significant advances in our understanding of these tumours. While these changes have been extremely positive in terms of surgical and systemic management, they have also created significant uncertainties concerning integration of local and locoregional radiotherapy into the therapeutic scheme. In parallel, radiotherapy itself has also experienced major advances. Beyond the evident technological advances, new radiobiological concepts have emerged, and genomic data and other patient-specific factors must now be integrated into individualized treatment approaches. In this context, “precision medicine” seeks to provide an answer to these open questions and uncertainties. Although precision medicine has been much discussed in the last five years or so, the concept remains somewhat ambiguous, and it often appear to be used as a “catch-all” term. The present review aims to clarify the meaning of this term and, more importantly, to critically evaluate the role and impact of precision medicine on breast cancer radiotherapy. Finally, we will discuss the current and future of precision medicine in radiotherapy.

## Introduction

**Figure 1:**
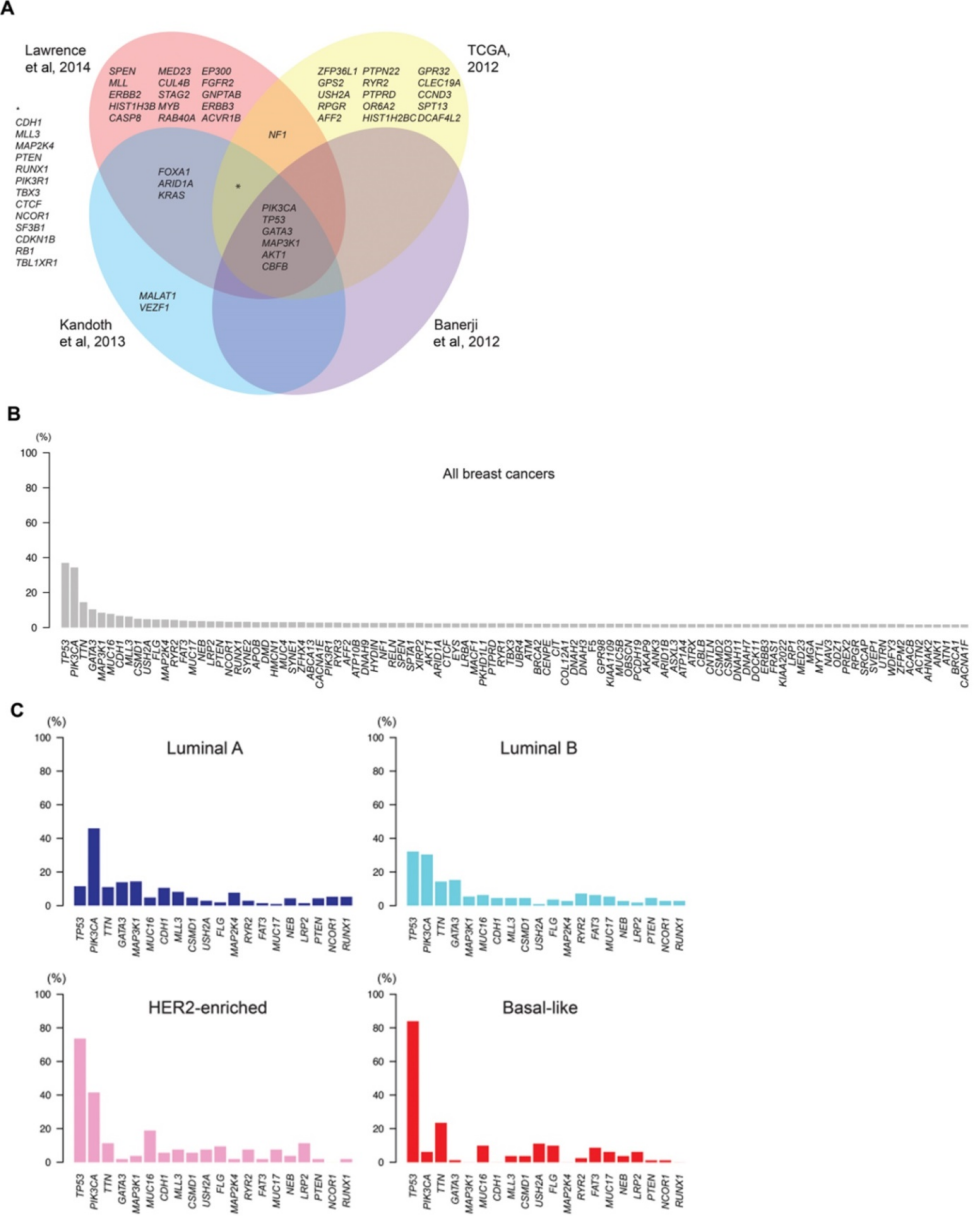
Intertumor genetic heterogeneity in breast cancer. At the genomic level, breast cancers are remarkably heterogeneous and no two tumors display an identical constellation of somatic mutations. **A)** Venn diagram illustrates the significantly mutated genes in breast cancer identified in different sequencing studies. **B)** Mutational frequencies of the 100 most frequently mutated genes in all breast cancers, illustrating the small number of genes highly recurrently mutated and a long “tail” of genes with low mutational frequency. **C)** The mutational frequencies of the 20 most frequently mutated genes in breast cancers of luminal A, luminal B, HER2-enriched and basal-like “intrinsic” subtypes. TCGA: The Cancer Genome Atlas 3.

Breast cancer is the most commonly diagnosed cancer in women worldwide 1. However, “breast cancer” is not, histologically-speaking, a homogeneous disease that can be easily classified according to prognostic criteria such as local, regional or distant disease extension (e.g., anatomical criteria). Rather, it is a complex pathology with a multifactorial etiology, and is highly heterogeneous in terms of its histological, molecular, and genomic characteristics 2. This heterogeneity poses a major challenge to determining the optimal therapeutic sequence, particularly given the significant advances - in the span of only a few years - in classification systems. Until relatively recently, the staging was strictly based on anatomical disease extension, whereas it now includes conventional biomarkers such as estrogen receptors (ER), progesterone receptors (PR), HER2, ki-67, and histological grade (**Table 1**), as well as the molecular and gene profile of the tumour itself. Moreover, ongoing advance in genomic analysis techniques, which are gradually being validated and incorporated into clinical decision-making, has led to a reclassification of breast tumours based on their intrinsic biological characteristics. As a result, breast cancer is now recognized as a set of different diseases, each with a specific profile in terms of prognosis and treatment response, as shown in **Figure 1** 3.

The emergence of multigenic molecular assays (or genetic platforms) in recent years has revolutionized the management of breast cancer 4. Initially, these assays were designed to provide prognostic and predictive data on response to chemotherapy in patients with positive hormone receptor expression and negative lymph nodes. However, over time, findings from validation studies have increasingly supported assay use in patients with positive nodes and low burden regional disease. Those same studies have also validated the capacity of these assays to predict the likelihood that a patient will benefit from chemotherapy 5, findings that have directly led to a reduction in the use of chemotherapy in low and intermediate-risk patients. These multiple developments now allow clinicians to determine which patients should receive systemic treatment more accurately. However, the impact on radiotherapy remains undefined, at least until results are reported from validation studies currently underway.

Adjuvant radiotherapy plays an essential role in the management of breast cancer, improving both locoregional control (preventing recurrences) and overall survival 6, 7. Notwithstanding these benefits, in recent years there has been a concerted effort to identify low-risk subgroups and subtypes with a good prognosis in which adjuvant radiotherapy could be omitted. These efforts are based on the premise that not all women benefit equally from radiotherapy, and that radiotherapy is unlikely to significantly improve survival in certain well-defined patient profiles 8, 9.

Moreover, the increasing use of neoadjuvant therapy, together with improved pathological response rates, suggests post-mastectomy radiation doses could be de-escalated or even omitted altogether 10. This concept is directly related to recent efforts to define patient subgroups likely to benefit from the omission of a specific intervention, to prevent the associated acute or chronic side effects in patients who are unlikely to benefit (or only marginally benefit) from the treatment. The use of such treatments would be reserved for patients for whom there is a clear benefit. However, this approach overlooks the fact that we live in an era when, thanks to technological advances, it is possible to precisely deliver the radiation dose to the target volume while minimizing the dose to adjacent healthy organs and tissues. These characteristics of advanced, modern radiotherapy techniques also form part of “precision medicine”, which is evident in two aspects of radiation oncology: 1) technologically-driven improvements in radiation delivery and 2) individualized treatment, with a personalized prescription for the dose, fractionation scheme, and target volumes based on the patient's clinical and biological parameters 11.

## Definition of the term “precision medicine”

The term “precision medicine” has evolved from “personalized medicine”, the much discussed “Holy Grail” of medicine. Personalized medicine is based on the determination of the unique genetic and molecular profile of an individual with a specific disease, data which can be used to develop a fully individualized treatment plan 12. Over the years, particularly after the Human Genome Project was completed (due to the lack of immediate impact on health care), it became necessary to redefine the term “personalized medicine”, perhaps because the term “personalized” proved to be not only too ambitious, but also too restrictive. This term fails to account for a substantial amount of relevant information, most notably clinical parameters and environmental factors. Currently, the concept of personalised medicine has been practically limited to molecular medicine. By contrast, the term “precision medicine” is a much broader term, which does not refer to the development of specific drugs for each individual, but rather to the adaptation of medical practice to suit the specific characteristics of well-defined groups of individuals, by classifying them into homogeneous subgroups based on their susceptibility to a particular disease, their probability of responding to a given treatment, or other parameters. In this way, any intervention - ranging from preventive to highly complex treatments - could be targeted at the specific subpopulations most likely to benefit from that intervention, resulting in the more efficient management of resources and even a reduction in systemic inequities 13,14.

## Precision medicine applied to radiotherapy

**Figure 2:**
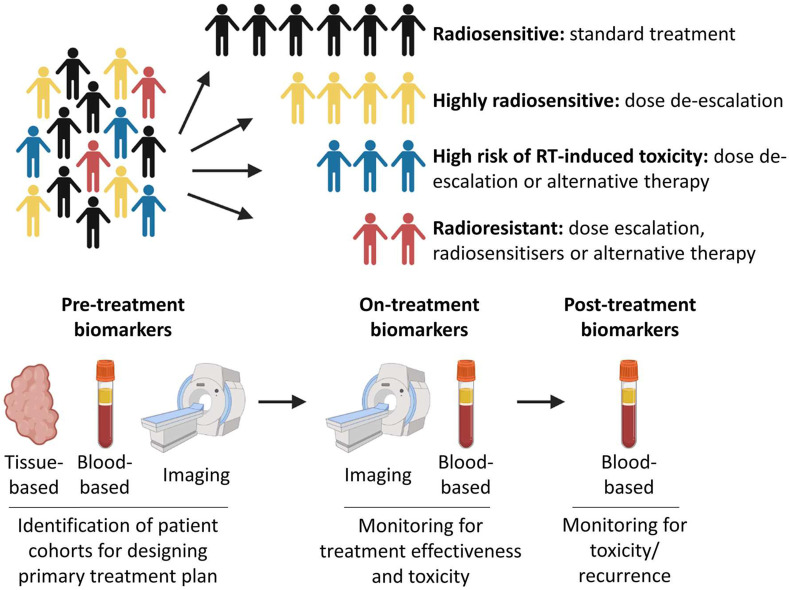
Precision medicine and radiotherapy. Patients could be stratified into different cohorts based on predicted intrinsic radiosensitivity and risk of toxicity. On-treatment monitoring may provide information on response to treatment, enabling adaptive changes to a patient's treatment to be made if necessary. Post-treatment biomarkers could be used to assess for evidence of toxicity, tumor recurrence or the development of metastatic disease 19.

Radiotherapy is a local, targeted treatment modality that delivers highly precise conformal doses of radiation to the target in order to induce cell death. Tumours and healthy tissues both have an intrinsic radiosensitivity whose response is influenced by the total dose and the fractionation scheme 15. Modern radiotherapy techniques can deliver conformal treatment with millimetric precision 16. In addition, treatment response can be optimized by using radiosensitizers 17. Radiotherapy may also play a role in modifying immune response 18, and some valuable bio-markers throughout the whole radiotherapy process were used to assess for evidence of toxicity, tumor recurrence or the development of the metastatic disease (**Figure 2**) 19. By these ways, the difference of breast cancers, which routinely are considered as only one kind disease, will be distinguished systematically.

For all these reasons, radiotherapy could be one of the critical areas for precision medicine, and, as we discuss in this review, it is likely to become increasingly relevant in the treatment of cancer shortly. Radiotherapy is one of the three pillars of cancer therapy; the large number of cases treated with radiation therapy is more than sufficient to conduct studies to establish prognostic and/or predictive subgroups and to validate the results. Once we have identified these subgroups, the results could be applied in two ways: 1) precision radiotherapy based on imaging and clinical characteristics or 2) precision radiotherapy based on radiobiology and genomics 20. The second point was discussed in-depth in this review.

## Genomically-guided radiotherapy

As discussed above, breast cancer is not a homogeneous entity, but rather a spectrum of related diseases grouped under one umbrella term, with each biological subtype having its own natural history and prognosis. Indeed, the discovery of the biological diversity in breast cancer has led to the re-stratification of patients into different prognostic groups. In turn, this has stimulated the development of systemic treatments directed at specific therapeutic targets, leading to improved survival and better control in appropriately selected patients. An excellent example of this is the development of anti-HER2 therapy in patients who overexpress this receptor. Over time, this biological subdivision of breast cancer has become increasingly relevant due to its predictive capacity, which can be used to predict the risk of metastatic progression and the probable impact of systemic treatment. Beyond this, multigene assays can also help to predict local or regional control 21.

Nguyen et al. 22 analyzed 793 breast cancer patients to evaluate the impact of molecular subtypes on local and regional control in patients treated with breast-conserving therapy followed by radiotherapy. Overall the probability of local recurrence in all subtypes was low, but particularly in the Luminal A subtype. Along the same lines, Caudle et al. 23 evaluated 595 patients treated with anthracycline and taxane-based neoadjuvant therapy followed by breast-conserving surgery (BCS) plus adjuvant radiotherapy. Although HER2+ patients were included, trastuzumab was not part of the neoadjuvant protocol. Locoregional recurrence (LRR)-free survival in patients with the HR+/HER2- and HR+/HER2+ subtypes was excellent, regardless of tumour response to neoadjuvant chemotherapy; by contrast, patients with the HR-/HER2 + and HR-/HER2- subtypes who had a poor response to neoadjuvant treatment had a lower LRR-free survival rate, thus confirming a direct association between pathological complete response (pCR) and LRR-free survival. These findings are consistent with other reports, including the study by Guarnieri et al. 24 and the I-SPY1 multicenter trial 25, both of which found a higher rate of complete responses to neoadjuvant treatment in ER(-) patients. Guarneri and colleagues concluded that pCR could be considered a prognostic factor for overall survival (OS) while the I-SPY1 trial reached the same conclusion concerning relapse-free survival (RFS). The question, however, is how to integrate these findings into the adjuvant radiotherapy treatment plan effectively.

### Genomic assays for breast cancer

Beyond the “histological” and immunohistochemical biomarkers conventionally used to classify patients into the subtypes described above, the real revolution that has taken place in recent years is the incorporation of genomic assays into routine clinical practice. These tests have redefined our understanding of the biology of breast cancer and its correlation with prognosis. Data from genomic assays even allow us to predict treatment response and the likely impact of different treatments.

These genomic tests are primarily used to assess the risk of progression, but a critical assessment of their applicability shows that they differ substantially in many ways, particularly concerning: the methodologies used to quantify gene expression; the genes evaluated; the clinical variables and pathologies included in the algorithm; risk group stratification; and whether testing is centralized or not. Therefore, even though these tests have been validated and proven to improve decision-making, it is important to keep in mind that two different assays, performed on the same patient, could yield contradictory results in terms of risk group assignment. 4 Nevertheless, these tests have demonstrated their clinical applicability, which has resulted in a dramatic decrease in chemotherapy, thereby reducing the cost of treatment and making it more cost-effective overall 26.

Numerous genomic tests are available, most commonly to identify patient subgroups in which adjuvant chemotherapy treatment can be omitted. In this review, we focus on the tests whose results could potentially influence the management of locoregional radiotherapy. Assays that do not address this question have been omitted from this review (**Tables 2 & 3**).

### Oncotype DX

The Oncotype DX 21 genomic test (OncotypeDX, Genomic Health, CA) was one of the first clinically validated molecular tests to provide a risk stratification model that could predict the benefit of adjuvant chemotherapy in patients with breast cancer. It was initially validated for patients with positive hormone receptors (HR) and negative lymph nodes 27, and later for women with positive lymph nodes with low burden axillary disease 28. The test analyses formalin-fixed and paraffin-embedded tumour tissue to determine the expression of 21 genes using real-time reverse polymerase chain transcription (RT-PCR). Of these 21 genes, 16 are cancer-related and five are reference genes for normalization. The 16 genes were selected from 250 candidate genes based on the correlation between gene expression and the risk of distant recurrence. Validation studies have shown that this test provides information - beyond the usual histological and clinical parameters (immunohistochemical, histological and TNM) - about the potential benefits of chemotherapy in a given patient, as well as predictive data about the risk of recurrence 29. This genomic test is now widely used, with a growing body of evidence showing that its use has significantly reduced the prescription of chemotherapy in breast cancer patients; moreover, there has also been an increase in the number of patients whose test results indicated a high risk of recurrence - despite the presence of good prognostic factors such as postmenopausal status, positive HR status, and negative nodes 30.

Given that locoregional recurrence is a prognostic factor for distant failure, several authors have attempted to determine the association between the Oncotype Dx recurrence score and the risk of locoregional failure. The Mamounas group was one of the first to explore this approach. Those authors evaluated node-negative, ER+ patients from the NSABP trials (B-14 and B-20). Mamounas et al. 31 included 895 and 355 patients, respectively, from the B-14 trial who received adjuvant tamoxifen or placebo; they also included 424 patients from the B-20 trial who were treated with chemotherapy and tamoxifen. To determine the impact of the Oncotype DX score, they correlated the recurrence score with the time to locoregional recurrence, finding a statistically significant association between the recurrence score in patients with node-negative, ER+ breast cancer who received tamoxifen. This finding is similar to the known association between the recurrence score and distant failure in the same group of patients. The same association was confirmed in patients (regardless of age), who underwent mastectomy alone without adjuvant radiotherapy (which was not indicated), suggesting that patients with this clinical profile who have a high-risk recurrence score could benefit from adjuvant radiotherapy. Despite these promising results, the clinical applicability of OncotypeDx in this context is still pending validation.

### Oncotype DX Breast DCIS Score test

Studies have shown that adjuvant radiotherapy after BCS in patients with ductal carcinoma *in situ* (DCIS) reduces the likelihood of local recurrence (both *in situ* and invasive) by 50% 32. However, a significant number of patients do not receive adjuvant radiotherapy - even though it is considered the standard of care - mainly due to treatment decisions based on subjective, non-evidence based clinical criteria. Moreover, in patients with DCIS, there may be subgroups suitable for breast-conserving therapy without adjuvant radiotherapy, although this is not currently an option under current treatment standards.

Oncotype DCIS is a prognostic test to estimate the risk of local recurrence. The platform combines genomic data with clinical parameters (age, tumour size, surgical margins, and multifocality). The hypothesis underlying the development of this platform is that the Oncotype DX recurrence score could provide the same information in patients diagnosed with DCIS. It was first clinically validated in formalin-fixed, paraffin-embedded histological archival specimen blocks, with or without an invasive component, obtained from patients with DCIS (treated with BCS without radiotherapy) included in the E5194 ECOG-ACRIN clinical trial 33. Of the 670 randomized patients (DCIS grade [G]1 or G2 ≤ 2.5 cm versus G3 < 1 cm, and minimum surgical margin of 3 mm), tissue samples were available from 327 patients. In this sample, the expression of a panel of 12 genes (seven cancer-related genes and five reference genes) was determined. These 12 genes were originally identified in a previous study of five experimental datasets (none with samples from this trial), which was used as a validation cohort (i.e., not experimental). The 10-year risk of ipsilateral breast cancer for patients classified as low, intermediate, and high risk, was, respectively, 10.6%, 26.7%, and 25.9% (*p*=0.006). The risk of an invasive event at 10 years was 7%, 12.3% and 19.2%, respectively (*p*=0.003). On the multivariate analysis, the Oncotype DCIS recurrence score, tumour size, and postmenopausal status were all significantly associated with local recurrence of any type 34.

This assay was independently validated in a subgroup of 3,303 patients from the Canadian cohort study (Ontario) involving 3,762 patients diagnosed with pure DCIS; histological samples from 80% of patients were available for centralized review 35. That study showed that the Oncotype DCIS recurrence score was significantly associated with the risk of ipsilateral breast recurrence in ER+ patients [hazard ratio (HR), 2.3; 95% CI, 1.41-3.59; *p*<0.001] and in the full cohort (including both ER positive and negative patients, 95% of whom were ER+) (HR=2.2; 95% CI, 1.43-3.22; *p*<0.001). The 10-year risk of developing an ipsilateral breast event was, respectively, 12.7%, 33%, and 27.8% for patients with low, intermediate, or high risk. The corresponding 10-year risk of an invasive event was 8%, 20.9%, and 15.5%, respectively 36. The oncological outcomes in these patients are worth highlighting. Two subgroups were evaluated: 571 patients who underwent BCS alone, and 689 who underwent BCS plus adjuvant radiotherapy. At a median follow up of 9.2 years, 100 local recurrences were registered (57 invasives, 44 DCIS) in the first group versus 86 in the latter group (adjuvant radiotherapy). These results are of interest because, not only was the recurrence rate lower in the group that received adjuvant radiotherapy, but those patients also presented clinical factors at diagnosis suggestive of a worse prognosis. On the multivariate analysis, a propensity-score analysis showed that the following factors were significantly associated with local recurrence: radiotherapy, age at diagnosis, tumour size, and multifocality. The Oncotype DX DCIS risk group classification, adjusted for these variables, was significantly associated with the risk of local recurrence; however, even though women with a high-risk score had a higher risk of local recurrence (both DCIS and invasive), and also a more significant absolute benefit from radiotherapy compared to the low-risk group, the model was not a statistically significant predictor of the benefit of radiotherapy 37.

Given these findings, it is clear it would be risky to omit adjuvant radiotherapy after BCS in patients with DCIS based exclusively on data provided by this genomic assay. Nevertheless, the DCIS score has altered clinical practice-even impacting recommendations for adjuvant radiotherapy in some cohorts 38-although more robust data from clinical trials are needed to validate the role of this assay to guide radiotherapy administration in women with breast cancer (NCT02766881). In short, the impact of omitting adjuvant radiotherapy on oncological and clinical outcomes remains unknown at present. Nonetheless, it is important to emphasise that this assay was not designed, nor has it been validated, as a predictor of response to radiotherapy or hormone therapy. Moreover, the data reported to date have failed to identify a subgroup of patients who would not benefit from adjuvant radiotherapy after BCS. Finally, this genomic test has not been evaluated to determine the existence of high-risk DCIS subgroups that could benefit from post-mastectomy radiotherapy despite the presence of good prognostic clinical factors 39.

### Prosigna/PAM 50

The classification of breast cancer according to the biological subtype has gained prominence in recent years. At the same time, due to technological innovations, it is now possible to identify transcriptional differences based on RT-PCR and microarray analyses, correlating biological subtypes with different transcriptional patterns. In the year 2000, Perou and colleagues classified, for the first time, breast cancer at the molecular level. Those authors hypothesised that phenotypic diversity in breast cancer was attributable to the diversity in gene expression patterns, which could be studied using cDNA microarrays 40,41. They evaluated 65 surgically-resected specimens (normal and cancerous tissues) from 42 women with breast cancer who underwent neoadjuvant treatment. They identified 496 genes that presented more significant variability in terms of their expression among the tumours, but their expression was only minimally variable in the samples from the same patient (intrinsic genes). Based on these data, together with microarray and RT-PCR techniques, Parker et al. developed a simplified signature for a 50-gene subtype predictor (classifier) and five control genes that were strongly correlated with the biological subtypes 42 and superior to routine immunohistochemical studies 43. This gene signature assay (PAM50) is marketed as the Prosigna-Breast Cancer Prognostic Gene Signature Assay. The algorithm resulting from the combination of genomic and clinical data (proliferative index, tumour size, nodal involvement) provides a Risk of Recurrence (ROR) score that assigns the patient to one of three risk categories (low, intermediate, high) associated with varying probabilities of developing a distant recurrence at 10 years. This assay was validated on patient samples from various clinical trials. An analysis of 1017 patients in the ATAC study showed that the PAM50 ROR in ER+, node-negative patients was more accurate than the OncotypeDx recurrence score in identifying high-risk patients, reducing the number of intermediate-risk patients 44. Besides, an analysis of 1478 ER+ postmenopausal patients from the ABCSG-8 trial (tamoxifen vs tamoxifen followed by adjuvant anastrozole) showed that the ROR had a better prognostic capacity than conventional clinical parameters, leading the authors to conclude that adjuvant chemotherapy in low-risk patients did not provide any expected oncological benefits 45.

The PAM50 assay is also being studied to determine the impact of the different intrinsic biological subtypes on local recurrences, and to identify clinical cases or intrinsic subtypes in which adjuvant radiotherapy can be omitted. A secondary analysis of the ABSCG-8 trial examined the association between the ROR and the biological subtype in terms of LRR-free survival, concluding that the ROR was highly predictive of locoregional recurrence, regardless of nodal status, age, or tumour size 46. That study included 1,308 patients, 79% of whom were treated with BCS plus adjuvant radiotherapy. At 10 years, a total of 34 locoregional recurrences were observed. A comparison of outcomes by biological subtype revealed a significant difference (*p*=0.022) in survival free of local or nodal recurrence between luminal A (98.1%) and luminal B (95.9%). However, that study was not designed for decision-making for adjuvant radiotherapy, which provides an opportunity for other researchers to evaluate this question. The PRECISION (Profiling Early Breast Cancer for Radiotherapy Omission) trial is a non-randomized, phase II trial (NCT02653755), which began patient recruitment in 2016. That trial aims to evaluate the omission of breast radiotherapy action after lumpectomy in patients with low-risk (ROR score) breast cancer in whom adjuvant endocrine therapy is indicated. A total of 1,380 patients are expected to be included in that trial through the year 2023. Along the same lines, the EXPERT trial (Examining Personalised Radiotherapy for Low-risk Early Breast Cancer), with similar inclusion criteria and objectives, is being carried out by the Breast Cancer Trial Group in Australia and New Zealand (ANZ 1601/BIG16-02) to identify women (using Prosigna) with low-risk breast cancer. Recruitment is expected to be completed in 2022 [TROG 16.04, ANZ 1601/BIG16-02].

### Danish breast cancer cooperative group gene profile

The study was based on the patient cohort included in the Danish Breast Cancer Cooperative Group (DBCG82bc) trial 47. The objective was to validate a gene profile associated with the risk of locoregional recurrence in patients who did not receive adjuvant radiotherapy. A second aim was to determine if this profile could predict the benefit of radiotherapy in patients with high-risk breast cancer who received adjuvant systemic treatment and were randomized to receive or not postmastectomy radiotherapy. Gene expression analysis was performed on frozen tumour tissue samples obtained from 191 patients, all of whom underwent mastectomy. The genes were identified by the Lasso method, and the endpoint was locoregional recurrence (LRR). A weighted gene expression index (DBCG-RT profile) was calculated and transferred to quantitative real-time PCR (RT-PCR) in formalin-fixed and paraffin-embedded samples (FFPE). Seven genes were identified; based on the DBCG-RT profile, the 191 patients were categorized as having either high or low risk of LRR. A “low risk” classification was consistently associated with biological subtypes (Luminal A) indicative of good prognosis and these patients also had better locoregional control; by contrast, the “high risk” category was correlated with triple negative and HER2 subtypes. Based on these findings, it is clear that this gene profile strongly identifies the same subtypes, with the same prognostic behavior, as if they had been determined by other methods (histological/immunohistochemical). Adjuvant radiotherapy after mastectomy significantly reduced the risk of recurrence in patients with a “high risk” of LRR, but not in “low risk” patients. Interestingly, both risk groups (high and low) were present in all clinicopathological subgroups, and their outcomes were independent of classic variables such as TNM. For example, among the patients considered to present a high risk of recurrence according to conventional criteria (tumour size>5 cm in ≥4 positive lymph nodes), some were considered to have a very low risk of LRR based on the DBCG-RT profile; conversely, some patients with low lymph node load or tumour size < 2 cm were classified as “high risk” (DBCG-RT profile), and radiotherapy was beneficial in those cases. These findings suggest that the DBCG-RT profile could add valuable information to help guide decision-making 48.

## The possibility of applying biology-guided radiation therapy

In recent years, it seems that the vast majority of research has been centred on identifying patient subsets in which adjuvant radiotherapy can be safely omitted. In the coming years, it is certainly possible that a genomic assay capable of identifying this patient subset will be developed and validated. However, a reasonable criticism of the current role of biomarkers in radiation oncology is the lack of specific biomarkers to guide the indication for radiotherapy treatment itself. In general, biomarkers evaluated in the past were not selected (nor evaluated) for their specificity as indicators for radiotherapy. Indeed, most of the biomarkers shown to correlate with post-radiotherapy treatment outcomes are also prognostic in patients who do not undergo radiotherapy. In this regard, rather than using genomics to identify biomarkers to guide the omission of radiotherapy in low-risk patients, the real benefit would be to use genomics to adapt the treatment approach to suit the intrinsic characteristics of the tumour itself.

Eschrich et al. developed and validated the radiation sensitivity index (RSI) as a predictor of the inherent radiosensitivity of a given tumour. This indicator was evaluated in different cell lines from different types of cancer (including breast cancer) 49, and validated on datasets from two independent centres, the Karolinska University Hospital (n=159) and the Erasmus Medical Center (n=344). In the first cohort (Karolinska) 50, patients whose tumours were predicted to be radiosensitive had better 5-year RFS outcomes than those with radiation-resistant tumours (95% vs 75%, *p*=0.0212), but there were no differences between patients who did not undergo radiotherapy (71% vs 77%, *p*=0.6744), thus validating RSI in this patient subset, a finding that indicates that RSI is specific to radiotherapy. In the Erasmus cohort, radiosensitive patients treated with radiotherapy had better 5-year metastasis-free survival than patients with radiation-resistant tumours (77% vs 64%, *p*=0.0409), with no differences in patients who did not receive RT (80% vs 81%, *p*=0.9425). The multivariate analysis showed that the variable that had the strongest influence on outcomes in radiotherapy-treated patients was RSI, demonstrating that RSI is an independent predictor of outcomes in ER+ patients treated with radiotherapy. Subsequently, Sjöström et al. 51 developed and validated a gene expression assay to predict the risk of local breast cancer recurrence and its response to adjuvant radiotherapy after BCS. Torres-Roca and colleagues evaluated the possibility of integrating RSI with molecular subtype data to identify patient subgroups with a higher risk of local recurrence after BCS+ radiotherapy, finding that patients with the triple negative and radioresistant subtype had an increased risk of local recurrence. These findings are sure to motivate more research in this area. The authors have suggested that the combination of RSI and molecular subtype could help to guide indications for adjuvant radiation therapy in breast cancer 52.

In recent years, numerous authors have attempted to integrate data on the intrinsic radiosensitivity of tumours with the traditional linear quadratic model. The conventional model quantifies the biological effect of the radiation dose on both the tumour and healthy tissue, but this model has a significant limitation: it is not patient specific, and its value is reduced when extreme hypofractionation is used 53. Integration would require developing a customized model using a patient-specific “α” to quantify the response of a specific tumour to radiotherapy to obtain a genomic-adjusted dose (GARD) 54. The premise underlying this model in breast cancer is that a significant number of radiosensitive women could be treated with a lower than standard dose without compromising local control. In addition, it could be possible to identify those patients in whom dose escalation would likely yield better results, thus offering “true” oncological personalization, as well as optimizing fractionation on a case by case basis 55. Ahmed et al. evaluated the feasibility of combining the linear quadratic model with the RSI, calculating the GARD that would optimize the individualised radiation dose for patients from two cohorts with triple negative (TN) breast cancer. Those authors also demonstrated that the GARD, like the RIS, is associated with the risk of local recurrence. Thus, they identified two groups of patients based on the RIS value. One group (RIS, 0.43-0.49) would likely benefit from dose escalation to 50-60 Gy (29% of the sample) and a second group (RIS up to 0.55, 13% of the sample) would benefit from higher doses (70Gy), in both cases with conventional fractionation schemes (2 Gy/session). Although the results of this hypothesis-generating study cannot be applied to routine clinical practice, these findings warrant a prospective clinical trial 56.

While the intrinsic radiosensitivity of the tumour is important, the radiosensitivity of healthy tissues is also relevant. In fact, many studies have explored the impact of radiation on healthy tissues and the likelihood of developing specific side effects. In breast cancer, manifestations of acute and chronic toxicity - fibrosis, edema, telangiectasias, hyperpigmentation, and different degrees of dermitis - have been associated with clinical risk factors (tobacco use, body mass index, hypertension, skin quality prior to radiotherapy) 57 or with the radiotherapy treatment itself (outdated techniques, size of the volume irradiated, and unfavourable dose-volume histograms) 58. However, treatment-related toxicity has not been eliminated, even in young patients with no relevant risk factors treated with state-of-the-art techniques such as hypofractionated IMRT or partial breast irradiation 59,60. For healthy tissues - at least in some organs - the gene profile could influence up to 80% of radiosensitivity and, therefore, the associated toxicity 61. That said, validation studies are still lacking in this field, and most of the studies published to date present methodological deficiencies, which limits reproducibility 62. Genome-wide association studies (GWAS) have been conducted to identify common breast cancer susceptibility alleles. These studies seek to expand the search for specific candidate genes to the study of a large number of single-nucleotide polymorphisms (SNP) in genes that encode proteins involved in pathways associated with inflammatory processes in tissues and with DNA damage, to find consistent differences between these SNPs in order to determine the association between these SNPs and radiation side effects 63. Conceivably, if a large number of SNPs can be identified, then it would be possible to establish a polygenic risk score that could subsequently be applied in routine clinical practice 64. GWAS technology is based on genotyping platforms (chip-based microarray technology) that can evaluate hundreds to thousands of SNPs simultaneously. However, to apply this in clinical practice, well-structured and homogeneous clinical data are needed to avoid bias 65, but this is difficult given that most analyses have been conducted in small cohorts, which substantially lowers the statistical power 66. Even though some published studies have confirmed these associations, few have been independently validated and nearly all suffer from a limited “n” 67. International collaborative groups have been formed to expand our understanding of the genomic basis for differences in radiosensitivity 68 by generating large, well-structured datasets that can be evaluated and associated with clinical characteristics 69, as well as to establish uniform working protocols (methodologies) and to jointly communicate the results of these genetic association studies in radiogenomics 70.

In recent years, there has been a geometric increase in the number of published studies on breast cancer. Unfortunately, the quality of the evidence is limited. Consequently, we must remain cautious, avoiding categorical statements, and proceeding carefully when seeking to apply discoveries into routine clinical practice. Nonetheless, several interesting studies have been published in recent years, and the promising results reported in those studies open the door to further research in this field. Mutations in the ATM (ataxia-telangiectasia mutated) gene were among the first to be evaluated for their possible association with tissue radiosensitivity. The ATM gene plays an important role in response to ionizing radiation and it is involved in the detection of DNA double-strand breaks and the initiation of pathways leading to the arrest of the DNA repair cycle or apoptosis 71. It has been shown that patients with a truncated mutation in both copies of the ATM gene are likely to develop significant radiation-induced toxicity, and it is precise that these patients who develop ataxia-telangiectasia syndrome 72. The SNP rs1801516 (c.5557G> A, p.Asp1853Asn) is among the ATM SNPs that have been most thoroughly investigated. Its impact on radiation-induced toxicity has been evaluated in three meta-analyses, with conflicting results: two of those studies found a direct association with an increased probability of fibrosis 73 and acute toxicity 74, but the third did not identify any consistent associations 75. Andreassen et al. 76, on behalf of the International Radiogenomics Consortium, conducted an individual patient data meta-analysis to assess the relationship between the ATM rs1801516 SNP and toxicity after radiotherapy in 5456 breast and prostate cancer patients from 17 different cohorts (breast cancer, n=2759; prostate cancer, n=2697), concluding that there is an association between the ATM rs1801516 Asn allele and increased risk of radiation-induced toxicity (odds ratio of approximately 1.5 for acute toxicity and 1.2 for late toxicity). These findings warrant future large studies in more homogeneous cohorts. Studies in smaller cohorts have also attempted to find associations between SNPs and post-radiotherapy pain 77, skin toxicity 78, and fibrosis 79, but the results have not been validated and further studies are required.

## Chances of precision medicine in breast cancer radiotherapy

Until just a few years ago, radiotherapy treatment in breast cancer was based on clinical parameters, in which the following questions were crucial: What is the size of the tumour? Is the patient node positive or not? Are the surgical margins negative? Mastectomy or BCS? Will the patient benefit from a boost or not? The responses to these questions determined the indication (or not) for radiotherapy, and also determined target volume selection and the scheme, usually 50 Gy in 25 sessions of 2 Gy. However, about 15 years ago, research efforts began to look beyond these basic questions. In parallel with technological advancements and an improved understanding of the radiobiology of both cancerous and healthy tissue, hypofractionated protocols began to be tested, generally by reducing the number of fractions from 25-30 sessions (the traditional scheme) to only 15-20 sessions. These approaches were evaluated in high quality, methodologically-sound randomized clinical trials, which confirmed the therapeutic equivalence of these hypofractionated protocols with conventional protocols, but with several potential advantages, including reduced costs, improved QoL, and less objective toxicity 80. From there, the next logical step was to propose partial breast irradiation protocols 81 and the omission of radiotherapy in selected patients 82. This began a period in which the focus shifted to treatment de-escalation, the concept of “less is more”, which evolved in parallel with surgical de-escalation, during which radical surgery (e.g., Halsted mastectomy) was abandoned in favour of less radical approaches, eventually leading to oncoplastic surgery 83. Furthermore, thanks to the development of new drugs and systemic treatments that have substantially improved locoregional tumour response, adjuvant radiotherapy is now even considered in certain node positive patients who respond to neoadjuvant chemotherapy 84. However, it is important to keep in mind that all these advances are solely and exclusively based on clinical parameters, without consideration of the intrinsic biological characteristics present in individual tumours. In addition, these advances also ignore all of the new instruments capable of estimating the risk of recurrence and survival, as well as tools that use genomic data to estimate radiosensitivity and toxicity.

Although we are making progress (**Table 4**), this research is still in its infancy. Molecular signatures and radiosensitivity indices are still in the early stages of development, with limited clinical applicability due to the lack of validation in patient cohorts without selection biases (most validation studies have been performed on non-randomized datasets with numerous confounders). Moreover, currently available genomic assays to predict recurrence risk were not designed - and thus not validated to personalize radiotherapy (i.e., to determine whether radiotherapy is indicated or not and to define the target irradiation volumes) 85.

## Conclusions

Beyond the obvious technological and clinical advances (e.g., fractionation protocols) that have become standard practice in recent years, the true promise of precision radiotherapy in breast cancer is the integration of clinical and imaging data together with molecular data and genomic markers. This is expected to lead to the development of numerous prognostic and predictive instruments to help predict various outcomes, most importantly: a) treatment response based on the intrinsic radiosensitivity of the tumour, thus allowing for dose modification; b) the risk of locoregional recurrence, which could be used to guide target volume modifications; and c) the risk of acute or chronic toxicity, which would allow us to modify the treatment to avoid these undesired effects, or at least initiate early treatment, thus improving tolerance to radiotherapy.

At present, several uncertainties remain. The underlying mechanisms of individual radiosensitivity remain unclear, molecular assays that could potentially play a key role in radiotherapy decision-making still need to be validated, and quantification of a personalized risk index remains to be fully defined. Nevertheless, the ongoing progress in these areas - coupled with collaboration between centres to share structured, homogeneous data - provide plenty of reasons for optimism. In fact, we believe that the advances described in this review will place radiation oncology at the vanguard of precision medicine in breast cancer in coming years. As always, the overriding aim is to cure more patients and improve clinical outcomes.

## Figures and Tables

**Table 1:** Molecular subtypes of breast cancer

Subtype		Expression	Prognosis	Frequency
Luminal	Luminal A	ER+, PR -, HER2 -, ki 67 ≤ 14	Favourable	60%
	Luminal B	ER+, PR +, HER2-, ki67 > 14	Intermediate	
		ER+, PR-, HER2-		
Basal-like (triple negative)		ER-, PR-, HER2-	Poor	15-20%
HER2 +		HER2+	Poor, but targeted treatment is available	25%

ER: Estrogen receptors; PR: Progesterone receptors; HER2: Human epidermal receptor 2.

**Table 2:** Overview of the multigene platform assays and implications for treatment decision-making

	Oncotype DX	Oncotype DCIS	PAM50	Danish Breast Cancer Cooperative Group
Year	2004	2013	2009	2014
No. genes used	21	12	50	7
Method	RT-PCR	RT-PCR	RT-PCR	Lasso method transferred to RT-PCR
N. risk groups	3		3	2
Tissue type used	FFPE tissue samples	FFPE tissue samples	FFPE tissue samples	FFPE tissue samples
Risk categories	Low	Low	Low	Low
	Intermediate	High	Intermediate	High
	High		High	
Inclusion of clinical parameters	No	No	Yes	Yes
Indication for Testing^a^	ER+, HER2-, N- Estimates chemotherapy benefit and relapse risk during hormonotherapy.	DCIS	ER+/N- and N+ treated by hormonotherapy. Predicts 10-year metastasis-free survival.	High risk breast cancer treated with systemic therapy and mastectomy.
ASCO/NCCN^a^	Yes (strong)		Yes (moderate)	
Loco regional impact of the test	Predictor of loco regional relapse	Reveals the 10-year risk of local recurrence;	Predictor of loco regional relapse and local-regional free survival	Predictor of the benefit of radiotherapy
		Is significantly associated with risk of an invasive local recurrence;		
		Is significantly associated with risk of a DCIS local recurrence.		
Potential application in Radiotherapy	Potential predictor of the benefit of PMRT in high risk patients.	Omission of adjuvant radiotherapy after conservative surgery in low risk DCIS patients.	Omission of adjuvant radiotherapy after conservative surgery in low risk invasive breast cancer patients.	Potential predictor of the benefit of PMRT in high risk patients.
Validation studies	NSABP B14 (retrospective)	ECOG E5194 (prospective-retrospective)	ABCG8 (retrospective)	DBCG- 82bc trial (retrospective)
	NSABP B20 (retrospective)	Ontario DCIS cohort (retrospective)	ATAC (retrospective)	
	SWOG 8814 (retrospective)			
	TransATAC (retrospective)			
	TAILORx (prospective)			
	RxPonder (prospective)			

Abbreviations: DCIS: Ductal Carcinoma *In situ*; RT-PCR: Quantitative real-time PCR; DBCG-RT: Danish breast cancer group - Radiotherapy profile; ROR: Risk of recurrence; ^a^ASCO/NCCN recommendations: Molecular assays can be used to determine the benefits of early-stage breast cancer chemotherapy; FFPE: formalin-fixed, paraffin-embedded; PMRT: Post-mastectomyradiotherapy.

**Table 3:** Gene panels studied in each genomic assay

		Institution	Platform used	Type of trial	Objective	Study Start Date	Estimated enrolment	Estimated Study Completion Date
PRECISION	NCT02653755	Dana-Farber Cancer Institute	Prosigna	Interventional, non-randomized phase II clinical trial	Low risk score=omission of RT	May 2016	690	June 2023
TAILOR RT	NCT03488693	Canadian Cancer Trials Group	Oncotype DX	Interventional, randomized phase III clinical trial	Regional radiotherapy in Biomarker low risk node-positive breast cancer	May 2018	2140	December 2027
EXPERT	NCT02889874	Breast Cancer Trials, Australia and New Zealand	Prosigna	Interventional, randomized phase III clinical trial	Omission of radiation therapy in ROR ≤60	August 2017	1167	December 2023
RIGAIN	NCT04069884	Sun Yat-Sen Memorial Hospital of Sun Yat-Sen University	RecurIndex^a^	Interventional (Clinical Trial) Randomized Phase III	Avoidance of Regional Nodal Irradiation for Node Positive Breast Cancer	Not yet recruiting	1834	
BIORISE	NCT03252717	Institut du Cancer de Montpellier-Val d'Aurelle	Panel of five proteins: AK2- IDH2-ANX1- APEX1- HSC70	Interventional (Clinical Trial) Single Group Assignment.	To confirm the predictive value of the panel for radiation-induced late side effects after BCS+RT	August 2014	500 participants	August 2022

Search made on clinicaltrials.gov based on the following keywords: Radiotherapy, Recruiting, Not yet.recruiting Studies, Breast Cancer, Studies with Female Participants, Adult and excluding trials that did not consider the performance of a molecular, genomic or protein test. Abbreviations: RT, Radiotherapy; BCS, breast conserving surgery.

**Table 4:** Open clinical trials underway to evaluate the impact of data provided by gene or protein panels on radiotherapy treatment

	OncotypeDX	Oncotype DCIS	PAM 50			Danish Breast Cancer Cooperative Group Profile
Genes included	Ki-67	Ki67	BIRC5	CCNE1	TMEM45B	HLA-DQA
	STK15	STK15	CCNB1	SLC39A6	MDM2	RGS1
	Survivin	Survivin	CDC20	ACTR3B	ESR1	DNALI1
	CyclinB1	CyclinB1	MKI67	MAPT	KIF2C	hCG2023290
	MYBL2	MYBL2	PTTG1	MYC	FOXA1	IGKC
	ER	PR	EP55	SFRP1	PGR	OR8G2
	PR	GSTM1	TYMS	KRT14	ERBB2	ADH1B
	Bcl2		UBE2C	EXO1	GRB7	
	SCUBE2		FOXC1	KRT17	BCL2	
	Stromelysin3		CDC6	CENPF	FGFR4	
	Cathepsin L2		MIA	KRT5	EGFR	
	GBR7		KNTC2	CDCA1	BLVRA	
	HER2		UBE2T	MLPH	PHGDH	
	GSTM1		RRM2	MYBL2	BAG1	
	CD68		ANLN	MELK	CDH3	
	BAG 1		MMP11	CXXC5	NAT1	
			ORC6L	GPR160		
Reference genes	ACTB (β-actin)	ACTB (β-actin)				
	GAPDH	GAPDH	- 8 Housekeeping genes			
	RPLPO	RPLPO	- 6 Positive controls			
	GUS	GUS	- 8 Negative controls			
	TFRC	TFRC				

## Abbreviations

LRR: locoregional recurrence
RT-PCR: real-time PCR
RSI: radiation sensitivity index
ER: estrogen receptors
PR: progesterone receptors
HER2: human epidermal receptor 2
DCIS: ductal carcinoma *in situ*
ROR: risk of recurrence
FFPE: formalin-fixed, paraffin-embedded
PMRT: post-mastectomy radiotherapy
IMRT: intensity modulated radiation therapy
